# Neurological Manifestations of Systemic Lupus Erythematosus: A Comprehensive Review

**DOI:** 10.7759/cureus.79569

**Published:** 2025-02-24

**Authors:** Maleesha Jayasinghe, Fatemeh Rashidi, Ahmed Farid Gadelmawla, Jamir Pitton Rissardo, Masoumeh Rashidi, Christopher C Elendu, Ana Leticia Fornari Caprara, Ibrahim Khalil, Khalil I Hmedat, Mohamed Atef, Hania Moharam, Omesh Prathiraja

**Affiliations:** 1 Medicine, Nanjing Medical University, Nanjing, CHN; 2 Medicine, Faculty of Medicine, Menoufia University, Shibin Al Kawm, EGY; 3 Neurology, Cooper University Hospital, Camden, USA; 4 Neurology, Universidade Federal de Santa Maria, Santa Maria, BRA; 5 Neurological Surgery, Faculty of Medicine, Alexandria University, Alexandria, EGY; 6 Internal Medicine, Faculty of Medicine, Alexandria University, Alexandria, EGY; 7 Medicine, Assiut University, Assiut, EGY

**Keywords:** diagnosis of npsle, lupus erythematosus, neuropsychiatric systemic lupus erythematosus (npsle), sle, s: sle, systemic lupus erythematosus disease

## Abstract

Neurological involvement in systemic lupus erythematosus (SLE) poses significant challenges, impacting patient morbidity, mortality, and quality of life. This narrative review provides an update on the pathogenesis, clinical presentation, diagnosis, and management of neurological SLE. The multifaceted pathophysiology involves immune-mediated and vascular mechanisms such as autoantibodies, neuroinflammation, complement dysregulation, and genetic factors. Neuropsychiatric SLE (NPSLE) manifests in a variety of ways, including cognitive dysfunction, mood disorders, psychosis, cerebrovascular disease, demyelinating syndromes, and neuropathies. Diagnosing neurological SLE is complicated by nonspecific and fluctuating symptoms, requiring comprehensive neurological examination, neuroimaging, autoantibody profiling, and cerebrospinal fluid analysis. Current management strategies include corticosteroids, immunosuppressive agents, and emerging biologics targeting specific immune pathways. Managing neuropsychiatric symptoms, seizures, and neuropathic pain remains a complex aspect of treatment. This review highlights the importance of early recognition and tailored management approaches to improve patient outcomes in neurological SLE.

## Introduction and background

Systemic lupus erythematosus (SLE) is a chronic autoimmune disorder characterized by inflammation involving connective tissues. SLE typically tends to impact all organs and tissues in the body, and the specific symptoms experienced by patients might vary significantly. The exact cause of SLE is not fully understood. However, it is thought to arise from the complex interaction of genetic and hormonal factors and environmental exposures, which determine the occurrence of antibodies to nuclear and cytoplasmic antigens [1]. In addition, patients with SLE may have other autoantibodies, including anti-Scl-70 antibodies (found in systemic sclerosis), anti-La and anti-Ro antibodies (found in Sjogren's disease), anticardiolipin antibodies, and antiphospholipid antibodies (aPL antibodies), suggesting a broad connection between SLE and other autoimmune disorders [2].

The worldwide incidence of SLE varies between 1.5 and 11 cases per 100,000 person-years, whereas the prevalence ranges from 13 to 7,713.5 cases per 100,000 individuals [3-5]. The female gender predominance in SLE has been extensively documented, with a male-to-female patient ratio of 1:9. The occurrence and frequency of SLE in females frequently peak between the ages of 15 and 44 and 45 and 64, respectively [6]. SLE varies across all age groups but is more prevalent among those aged 15 to 45 years [7]. Research indicates that between 10% and 20% of individuals diagnosed with SLE develop the disease during childhood. Interestingly, patients with childhood-onset SLE have a higher prevalence of renal, neuropsychiatric, and cardiovascular complications than other groups [8-10].

A notable manifestation of the nervous system in SLE is neuropsychiatric SLE (NPSLE). The documented frequency of neurological symptoms associated with SLE varies from 14% to 95%, with a higher occurrence in children than adults [11,12]. It can impact the central and peripheral nervous systems (PNS) in various ways. The total prevalence rate was 56.3%, with a higher incidence in the central nervous system (CNS) at 93.1% compared to the PNS at 6.9% [13,14].

In 1999, the American College of Rheumatology (ACR) published a collection of NPSLE case definitions, which included 12 manifestations related to the CNS and seven manifestations associated with the PNS [15]. The CNS manifestations can be categorized into four psychiatric and eight neurological syndromes. They can also be classified as either focal (presenting as focal neurological deficits) or diffuse (including cognitive disorder, mood disorder, psychosis, acute confusional state, and anxiety disorder) [15]. However, a study of 128 participants uncovered a significant occurrence of NPSLE in this group of individuals with SLE who were not specifically chosen. The most prevalent NPSLE manifestations seen were headaches, cognitive impairment, and mental illnesses [16].

Physicians face difficulties diagnosing NPSLE due to the absence of specific and sensitive laboratory serum or CSF biomarkers, radiographic imaging abnormalities, and formal criteria for establishing the diagnosis and guiding therapy and management decisions in NPSLE [17]. NPSLE diagnosis is facilitated by many clinical, serological, immunological, electrophysiological, and neuroimaging tests [14]. Regrettably, even when healthcare personnel can recognize neurological or psychiatric disorders in patients with SLE, these patients are rarely subjected to thorough evaluation, resulting in a lack of diagnosis and treatment. This is because the required tests are demanding regarding resources and time [18]. Magro-Checa et al. introduced a diagnostic method for patients with NPSLE that relies on the clinical symptoms observed in patients with SLE [19]. Both patients with significant NPSLE and patients with moderate or non-NPSLE exhibited a higher rate of death compared to the general population [20]. A study revealed that people with NPSLE have a death rate that is 11 to 14 times higher than that of the general population. Interestingly, an acute confusional state was the most important indicator for predicting a negative outcome [20].

SLE, a complex autoimmune disease, can profoundly affect the nervous system, leading to a wide range of debilitating conditions that significantly affect patient outcomes. This narrative review presents the current information regarding NPSLE, focusing on the clinical symptoms, pathophysiological pathways that play a role in the condition, diagnostic challenges, and the current strategy for management and treatment.

## Review

Neurological involvement in SLE: proposed mechanisms

In patients with SLE, approximately one-third of neuropsychiatric events can be directly attributed to SLE, occurring in 21% of patients within the first 6.6 years of their illness [21]. CNS involvement is observed in 90% of cases, while the PNS accounts for only 10% [21]. Notably, there is extensive research on CNS disease in people with SLE, but the impact on the PNS is less well-established (Table 1). These studies have provided valuable insights into the range of CNS symptoms and the connection between certain CNS disorders and autoantibodies, immunologic markers, and elements of SLE activity [21,22]. However, only a few studies have explored the relationship between peripheral neuropathies, autoantibody patterns, and the characteristics of SLE activity [23].

**Table 1:** Immune-mediated mechanisms in neurological SLE SLE: systemic lupus erythematosus, aPL: antiphospholipid, NPSLE: neuropsychiatric systemic lupus erythematosus, CNS: central nervous system, MAP-2: microtubule-associated protein 2, AECA: anti-endothelial-cell antibody, β2-GPI: beta-2-glycoprotein I, anti-RP Ab: anti-ribosomal P protein antibodies, aPL Ab: antiphospholipid antibodies, anti-MAP-2 Ab: anti-microtubule-associated protein 2 antibodies, anti-GAPDH Ab: anti-glyceraldehyde-3-phosphate dehydrogenase antibodies, BBB: blood-brain barrier

References	Mechanism	Description	Autoantibodies	Manifestations
Bonfa et al. (1987) [24]	The anti-P antiidiotypic antibody population could induce disease by binding to membrane receptors, as has been shown with experimentally induced antibodies to insulin and acetylcholine	In 18 of 20 patients with psychosis secondary to SLE, autoantibodies to ribosomal P proteins were detected by immunoblotting and measured with a new radioimmunoassay using a synthetic peptide as antigen	Anti-RP Ab	Psychosis
Sanna et al. (2003) [25]	The strong association between aPL and neuropsychiatric manifestations in this study supports the theory that an occlusive vasculopathy may be a major mechanism for NPSLE	The study included 323 consecutive SLE patients and investigated the associations between aPL and neuropsychiatric manifestations	aPL Ab	Cerebrovascular disease, headache, and seizures
Williams et al. (2004) [26]	MAP-2 have repetitive microtubule-binding motifs so that they not only control cytoskeletal integrity but also interact with other structural elements of the cell	Sera from 100 patients with SLE, 74 patients with other neurological disorders and injuries, and 60 normal controls were examined both by enzyme immunoassays and by Western immunoblotting for autoantibodies to MAP-2	Anti-MAP-2 Ab	Psychosis, seizure, neuropathy, and cerebritis
Conti et al. (2004) [27]	It has been postulated that AECA reactivity may be due in part to the binding to a complex of β2-GPI with phospholipids on endothelial cells	This study included 51 unselected outpatients with SLE (44 women, 7 men; mean age 36.8 years, range 22–54 years; mean disease duration 9.4 years, range 0.5–26 years)	AECA	Psychosis and mood disturbances
Delunardo et al. (2016) [28]	Suggests that these antibodies, generated in the periphery, penetrate the CNS from the peripheral blood across the altered BBB and bind to cell surfaces, possibly interfering with neuronal function	Investigate the potential role of circulating autoantibodies specific to neuronal cell surface antigens in the pathophysiology of neuropsychiatric disorders	Anti-GAPDH Ab	Cognitive dysfunction

The CNS is affected by SLE and presents a wide range of neuropsychiatric symptoms. These symptoms include aseptic meningitis, cerebrovascular disease, demyelinating syndrome (DS), various types of headaches (such as migraine and benign intracranial hypertension), movement disorders, myelopathy, seizure disorders, acute confusion, anxiety disorders, and psychosis [15]. Although less common than CNS involvement, PNS involvement can still significantly impact patients. The signs include Guillain-Barré syndrome (GBS), autonomic dysfunction, mononeuropathy, myasthenia gravis, cranial neuropathy, plexopathy, and polyneuropathy [15].

Myelopathy, presented as acute transverse or longitudinal myelitis, is one of the less common neuropsychiatric manifestations of SLE [25,29-31]. SLE manifests as a spinal cord injury resulting in muscular paresis or paralysis and smooth muscle dysfunction, primarily in the form of sphincter dysfunction. The imaging method of choice in cases of suspected acute transverse myelitis (ATM) is magnetic resonance imaging (MRI) with intravenous contrast [32].

Symptoms of acute spinal cord injury in patients with myelitis in SLE, along with other neurological manifestations of SLE, are frequently accompanied by optic neuritis. Depression, dysmnesia, convulsions, psychosis, and ophthalmoplegia can also be mentioned as less frequent symptoms [29,31,33]. Additionally, some cases of single ATM associated with aseptic cerebrospinal meningitis have been reported [33-35]. The prognosis may involve full recovery, partial remission, disease progression arrest, or death. Early initiation of immunosuppressive therapy with intravenous cyclophosphamide and high doses of glucocorticosteroids, followed by oral glucocorticosteroids, can improve the long-term prognosis for patients [29,36-38].

The autoimmune/inflammatory pathway involves activating pro-inflammatory substances or producing autoantibodies that target neuronal cells, leading to damage by forming intrathecal immune complexes and disrupting the blood-brain barrier (BBB). Prior research has commonly suggested that increased BBB permeability significantly contributes to the development of NPSLE [39].

The complement system plays a crucial role in the inflammatory pathway, particularly in disrupting the integrity of the BBB, mainly through the action of complement C5a/C5aR. In vitro experiments have shown that C5a triggers endothelial cell death, most likely by binding to the C5aR1 receptor. It has been observed that microglia have high levels of C5aR1 receptor mRNA [40]. Endothelial cells, stimulated by immune complexes, produce pro-inflammatory cytokines and cellular adhesion molecules by activating NF-κB signaling. Jacob et al. investigated the impact of C5a/CD88 signaling on BBB integrity in SLE through the NF-κB pathway. The authors revealed an increase in NF-κB translocation. A subsequent study by the same group showed decreased levels of zona occludens but increased levels of microtubule-associated protein (MAP), which may contribute to increased BBB permeability [41]. Therefore, elevated systemic signaling, mediated by cytokines and complement, is the primary factor triggering apoptosis in CNS endothelial cells, resulting in BBB damage and increased permeability. The involvement of the TWEAK/Fn14 pathway is of particular significance [40]. Furthermore, diffuse NPSLE has been associated with anti-C1q and C3/AP50, while focal NPSLE is linked to C4 levels. This suggests that complement pathway activation may play a role in neurocognitive dysfunction in NPSLE [19].

Inflammatory mediators can disrupt the BBB, and at the same time, neuronal or glial cells can generate cytokines and chemokines within the intrathecal space, enhancing the BBB's permeability. Both processes could facilitate circulating autoantibodies and leukocyte infiltration into the CNS. The presence of inflammatory cytokines, including TNF-α, TWEAK, IFN-γ, IL-6, IL-8, and BAFF, has been observed in the cerebrospinal fluid of patients with NPSLE. This suggests that the acute inflammatory response plays a significant role in developing NPSLE [42]. Yoshio et al. discovered that the levels of certain cytokines (IL-6, IL-8, MCP-1/CCL2, IP-10/CXCL10, G-CSF, and GM-CSF) in the spinal fluid were not influenced by their levels in the blood of patients with central NPSLE. This suggests that these cytokines likely produce chemokines in the CNS, which are necessary to develop central NPSLE [43]. Other interleukins involved in the development of NPSLE include IL-1, IL-2, IL-10, APRIL, and IFN-⍺ [44]. Additionally, CCL-5 has been associated with NPSLE [44].

Previous research has shown strong evidence that IFN-α can lead to neuropsychiatric symptoms in both human and mouse models. Santer et al. propose that the serum and CSF of individuals with NPSLE may have abnormally high levels of IFN-α activity [45]. Furthermore, inhibiting IFN-α signaling decreases synaptic loss associated with microglia, improving anxiety-like behavior and cognitive impairments in 564Igi lupus-prone mice [46].

Among the documented cytokines, IL-6 is strongly correlated with NPSLE activity. Moreover, IL-6 in the CSF is a reliable indicator for diagnosing central NPSLE, with high sensitivity and specificity. A recent study has identified a more detailed categorization of NPSLE, observing that blood IL-6 and CSF IL-6 levels are significantly higher in cases of acute confusion states (ACS) compared to non-ACS diffuse NPSLE, which includes anxiety disorders, cognitive impairment, mood disorders, psychosis, and localized NPSLE. Furthermore, the Q albumin (CSF/serum albumin quotient) was significantly elevated in ACS compared to the other two categories of NPSLE. Interestingly, Q albumin correlates more strongly with serum IL-6 than CSF IL-6 in patients with diffuse NPSLE, including both ACS and non-ACS cases [47].

An overactive response of microglia in the brains of mice with SLE, specifically in MRL/MpJ-Faslpr/J mice (MRL-lpr; Strain 000485), was observed, indicating the presence of brain innate immunity. In NPSLE, the interaction between CD40 and hippocampal microglia plays a role in the progression of cognitive dysfunction. In mice, inhibiting microglial activation can reduce neuropsychiatric symptoms [48].

Autoantibodies in NPSLE

A prominent characteristic of SLE is the generation of autoantibodies, and many antibodies have been discovered to be linked to NPSLE symptoms. Thus far, 116 antibodies have been identified in SLE, with at least 20 linked to NPSLE, including 11 specifically targeting the brain and nine affecting the entire system [49].

aPL antibodies including β2-GPI, cardiolipin anticardiolipin, and lupus anticoagulant: aPL antibodies specifically bind to anionic phospholipids, such as β2-GPI, instead of being directed against these phospholipids, as their name might imply. These phospholipids are found in the plasma membrane and regulate the blood clotting cascade [50]. Procoagulant activation leads to thrombosis and cerebral infarction [51]. aPL antibodies are associated with focal and diffuse NPSLE symptoms, including cognitive dysfunction [52], seizures [53], and myelopathy [25].

Anti-RP antibodies: Anti-RP antibodies are serological indicators frequently seen in patients with SLE psychosis and may be associated with PNS complications [54]. Anti-RP antibodies are found near the carboxy-terminal end of the 60S subunit of ribosomes. They bind to three phosphorylated proteins, P0, P1, and P2 [55]. Anti-RP antibodies are thought to cross the BBB, enter neuronal cells, and hinder protein synthesis [56].

Anti-NMDA antibodies: The NMDA receptor is a glutamate receptor in the CNS that plays a crucial role in synaptic plasticity and memory formation [57]. The NR2A and NR2B subunits are present in the hippocampus, amygdala, and hypothalamus [58]. NMDA receptors consist of tetramers made up of NR1 subunits and two of the four NR2 (A-D) subunits [59]. Anti-NMDAR encephalitis is a neurological disorder caused by the immune system attacking the NMDAR receptors. There are reports of anti-NMDAR encephalitis in patients with SLE, but the exact mechanisms behind its development are not completely understood [60].

Anti-NMO-IgG/AQP4 antibodies: AQP4 is the primary protein facilitating water movement in the CNS. It is predominantly found at astrocytes' extremities and positioned at the BBB and the brain-cerebrospinal fluid barrier. AQP4 plays a crucial role in regulating the flow of water within cells [61]. Anti-AQP4 antibodies induce damage to astroglial cells by triggering an inflammatory immune response. This response activates complement-dependent cytotoxicity, which disrupts the BBB and leads to the infiltration of white blood cells and the release of cytokines. Consequently, this process causes damage to oligodendrocytes, myelin, and neurons [62]. Myelitis, antiphospholipid syndrome (APS), and AQP4-IgG positivity were observed in 6.7% of the patients with SLE [63].

Structural endothelial proteins (ECs): ECs are located on the inner linings of blood vessels and constitute a layer of cells known as the endothelium. ECs have not been previously recognized as constituents of the immune system. They are crucial in maintaining blood pressure and are involved in various physiological and pathological processes such as coagulation, fibrinolysis, angiogenesis, and immune cell activation [64]. In autoimmune disorders, regulating endothelial cells by the adaptive and innate immune systems is critical, as endothelial cells contribute to persistent inflammation through angiogenesis, immune cell recruitment, and antigen presentation [65]. EC dysfunction may lead to microvascular changes in the white matter of patients with SLE, resulting in vascular dementia features. Also, microvascular abnormalities in the peripheral nerves may contribute to neuropathy in SLE [66].

Anti-SBSN antibodies are induced by astrocyte exposure to antibodies that have altered senescence and autophagy pathways [67]. It can serve as a useful indicator to distinguish NPSLE from SLE without neuropsychiatric symptoms, as anti-SBSN antibodies and their related immune complex were found solely in the CSF of NPSLE [68].

Anti-endothelial cell antibodies (AECA): AECA are self-produced antibodies that specifically attack endothelial cells, which form the inner lining of blood vessels [69]. In the context of NPSLE, these antibodies have been linked to several disease-causing processes [66,70].

Anti-UCH-L1 antibodies: Li et al. revealed that anti-UCH-L1 antibodies can be a dependable biomarker in the CSF to diagnose NPSLE. Additionally, the concentrations of UCH-L1 in the CSF may serve as an indicator of the intensity of NPSLE [71]. Furthermore, this marker was linked to increased disease severity and overall disease activity [72]. Similarly, a recent study on autoantibodies found that the autoantibody UCH58-69, which targets amino acids 58-69 of UCH-L1, has high specificity and diagnostic significance in distinguishing NPSLE patients from SLE patients without neuropsychiatric symptoms. The study found that NPSLE patients had significantly elevated levels of anti-UCH58-69 in their serum compared to SLE patients without neuropsychiatric symptoms. Furthermore, these levels correlated with the condition's severity [69].

Anti-GAPDH antibodies: GAPDH has been identified as a new autoantigen linked to neuropsychiatric diseases. Delunardo et al. revealed a strong positive relationship between levels of anti-GAPDH antibodies in the blood and detrimental cognitive and mood conditions (such as schizophrenia and major depression) in patients with SLE. The levels of anti-GAPDH antibodies were elevated in SLE patients who had psychotic symptoms compared to SLE patients who did not have psychotic symptoms [28]. Anti-GAPDH antibodies can induce neurite interaction and impair neuronal plasticity by blocking and binding synaptic molecules. These antibody levels are increased in NPSLE and are associated with cognitive dysfunction and psychiatric manifestations [73].

Anti-TPI antibodies: TPI is an enzyme that converts dihydroxyacetone phosphate to glyceraldehyde-3-phosphate. It is present in neurons and red blood cells [74]. Anti-TPI antibodies have been linked to NPSLE, mainly showing a greater occurrence of aseptic meningitis and higher levels of serum IgG in NPSLE patients who test positive for anti-TPI antibodies compared to those who test negative for anti-TPI antibodies [75].

Anti-MAP-2 antibodies: MAP-2 is a cytoskeletal protein found in neuronal cells that plays a crucial role in initiating and stabilizing microtubules, regulating the movement of organelles and protein kinases involved in signal transduction [76]. Anti-MAP-2 antibodies are linked to damage and death of neurons and are notably increased in the CSF of individuals with NPSLE [26]. Neuropsychiatric symptoms, such as psychosis, schizophrenia, bipolar disorder, and major depression, have been linked to the presence of anti-MAP-2 antibodies [77].

Anti-U1RNP antibodies: Autoimmune disorders such as mixed connective tissue disease, systemic sclerosis, and SLE are associated with anti-U1RNP antibodies [78]. SnRNPs, or small nuclear ribonucleoproteins, are abundant RNA-protein complexes in the nucleus. They are crucial in processing pre-mRNA and other proteins that make up the spliceosome [79]. Anti-U1RNP antibodies are associated with an increased risk of NPSLE, mainly when detected in the CSF [80].

Others: Brain cytoplasmic RNA (BC RNA) refers to a type of non-coding RNA molecule, specifically BC200 RNA (also known as BC RNA 1 or BCYRN1), which is primarily found in the cytoplasm of neurons within the brain and plays a role in regulating protein translation by inhibiting translation initiation factors, essentially controlling gene expression at the post-transcriptional level. Anti-BC RNA induction decreases the delivery of BC RNA to synaptodendritic sites in the brain. The decrease of BC RNA in the CNS has already been associated with some types of NPSLE, mainly involving cognitive impairment [81].

Cerebrovascular Pathway

Cerebrovascular events frequently manifest in increased disease activity and evidence of illness-related tissue injury [82]. In NPSLE, the most widely accepted cause of cerebrovascular disease is thrombosis induced by aPL antibodies. Prominent risk factors for this condition include chronic and high disease activity, a high cumulative corticosteroid dosage, persistent positivity for aPL antibodies at moderate-to-high levels, heart valve disease, and systemic hypertension [83]. The prevalence of SLE infections ranges from 3% to 20%, with a potential contribution of up to 15% to overall mortality [84,85].

APS is an autoimmune disorder distinguished by defined aPL antibodies, namely anti-cardiolipin IgM and IgG, anti-beta-2 glycoprotein IgM and IgG, and lupus anticoagulant. These aPL antibodies induce a condition of hypercoagulability, resulting in the formation of venous and arterial thrombi. The presence of APS can manifest as a primary disease. Nevertheless, it is worth noting that approximately 20-30% of patients with SLE test positive for aPL antibodies [86]. Moreover, when aPL antibody levels reach moderate-to-high levels, patients are at an elevated risk of thrombosis, even without prior thrombotic episodes. aPL antibodies have been identified as agents involved in the vascular pathway of NPSLE pathogenesis, leading to various adverse outcomes such as strokes, venous thromboembolism, cerebral venous sinus thrombosis, cognitive impairment, peripheral neuropathy, and chorea. APS has been associated with many clinical manifestations, such as maternal health complications and livedoid vasculopathy [87]. The CNS exhibits a higher vulnerability to thrombus formation than other tissues in APS, which is believed to be associated with specific receptors in the brain vasculature [88].

Vasculitis is a group of illnesses characterized by inflammation in blood vessels of varying sizes, occasionally accompanied by fibrinoid necrosis and subsequent vessel wall loss. It is an infrequent manifestation of SLE, with a prevalence of less than 7% in patients with NPSLE [89]. Interestingly, CNS SLE vasculopathy is usually a non-inflammatory condition that impacts small arterioles and capillaries, forming micro-infarcts and hemorrhages [90]. Immune complexes can induce endothelial dysfunction by stimulating endothelial cells to express vascular cell adhesion molecule 1, thus facilitating the recruitment of monocytes into the arterial wall [91]. Anti-endothelial cell antibodies, generated in the context of SLE, elicit the production of proinflammatory factors and foster the adherence of monocytes to endothelial cells, resulting in the subsequent inflammation of vessel walls. The presence of anti-endothelial cell antibodies leads to an increased release of cytokines, including IL-1, IL-6, and IL-8. This physiological response ultimately results in endothelial death [92]. Anti-endothelial cell antibodies and complement significantly contribute to vasculitis development in medium-sized arteries. This condition can lead to various complications, including luminal stenosis, small-vessel noninflammatory vasculopathy, microvessel occlusion, multifocal microinfarcts, intracranial embolism, and microhemorrhages [13].

Hypertension, diabetes mellitus, cigarette smoking, dyslipidemia, and a sedentary lifestyle are considered both traditional and modifiable risk factors for stroke and cardiovascular diseases. Hypertension is more common in SLE and APS than in the general population, and it can be explained by chronic inflammation [92]. Additionally, systemic inflammatory processes have been identified as the underlying cause of early atherosclerosis, also known as accelerated atherosclerosis [93]. Other factors influencing atherosclerosis are lupus nephritis, steroid therapy, and vitamin D deficiency.

SLE patients who have previously experienced a transient ischemic attack (TIA) have a 57% chance of developing an ischemic stroke [94]. The incidence of stroke in SLE ranges from 2% to 19% [95,96]. The incidence of ischemic stroke is twice as high in patients with SLE than in the general population and encompasses elements beyond the conventional Framingham risk factors. Additional factors that contribute to the higher risk of an ischemic cerebrovascular event include the presence of vasculitis, associated APS and positive aPL autoantibodies (found in 9.4% of cases), endocarditis, Libman-Sacks endocarditis, hyperviscosity (increased homocysteine levels), genetic polymorphism (the presence of allele GT20), and hypertension [82]. Consistent with this observation, a research study revealed that males with β2-GPI-dependent IgG anticardiolipin antibodies had a 1.5-fold increased risk of stroke and acute myocardial infarction compared to those without such antibodies [16]. Additionally, another study indicated that elevated levels of anticardiolipin antibodies in the bloodstream, irrespective of other cardiovascular risk factors, were a significant predictor of future stroke and TIA in females but not in males [97].

Individuals with autoimmune disorders have a twofold increased risk of experiencing an ischemic stroke within the first year of hospitalization compared to those without hospitalization. Interestingly, the risk of having a stroke remains elevated for at least ten years after the first hospitalization [98]. Also, after the first ischemic stroke, the risk of having another stroke in ten years is as high as 35% [99].

Genetic and Environmental Factors

Numerous studies have proven the link between genetic factors and the development of SLE. Nevertheless, investigating the genes responsible for humans' susceptibility to SLE has proven formidable, mainly due to limited epistasis, genetic heterogeneity, ethnic heterogeneity, and environmental factors. Determining the specific genotypes that contribute to NPSLE pathophysiology remains elusive. A meta-analysis found that the Fc³RIIIa, Fc³RIIIb, and ITGAM genotypes have been identified as probable susceptibility genes for NPSLE following a comprehensive examination of genetic variants associated with this disorder [100].

Specifically, it has been observed that patients with NPSLE have mutations in TREX1, a gene responsible for encoding DNase III [101]. Furthermore, variations in this gene have been linked to the presentation of CNS involvement, including seizures [102]. There is evidence suggesting that the HLA-DRB1*04/*13 genotype and the single nucleotide polymorphism (SNP) rs10181656 in STAT4, which encodes the signal transducer and activator of transcription 4, are linked to strokes in individuals with SLE [103]. This association remains significant even after controlling for aPL antibody status and conventional cardiovascular risk factors [104,105]. A comprehensive assessment has been conducted on the collective impact of SNPs in multiple genes linked to SLE, namely HLADRB1, IRF5, STAT4, BLK, TNFAIP3, TNIP1, FCGR2B, and TNFSF13, in Japanese individuals diagnosed with SLE. The presence of ten or more SNPs doubled the likelihood of neurological symptoms in these patients [106].

The induction or exacerbation of autoimmune diseases, such as SLE, by infection can be attributed to various mechanisms, including molecular mimicry, epitope spreading, polyclonal activation of B-cells, bystander expansion of auto-reactive T-cells, and viral and bacterial super-antigens [107]. The clinical presentations observed in NPSLE result from immunological dysregulation, characterized by the development of autoantibodies or vaso-occlusive events associated with the presence of aPL antibodies. Disease-associated immunological dysfunction and the immunosuppressive effects of treatment contribute to an increased vulnerability to infection [108]. Furthermore, the bimodal survival pattern in SLE has been reevaluated, revealing that death resulting from infections persisted throughout the progression of the disease [109].

The prevalence of CNS involvement in patients with SLE is estimated to range from 18% to 67% [110]. In SLE, infections contribute to around 20-55% of morbidity and mortality [111]. The CNS is accountable for 3% of these infections [111]. However, a significant proportion of NPSLE patients, up to 81%, do not exhibit any accompanying systemic SLE activity [110]. The predominant pathogens are *Mycobacterium tuberculosis* and *Cryptococcus neoformans* [112]. Notwithstanding their infrequent occurrence, CNS infections result in significant morbidity and mortality [113]. The results of the correlation study revealed a subtle positive relationship between rubella IgM antibody titers and the presence of psychosis in individuals with NPSLE [114].

The ultraviolet (UV) radiation emitted by sunlight is typically categorized into three broad bands: UV-C (200-290 nm: distant UV, germicidal UV), UV-B (290-320 nm: midrange UV, sunburn radiation), and UV-A (320-400 nm: near UV, black light). It is observed that the stratospheric ozone layer fully absorbs UV-C light, but UV-B radiation is only partially absorbed. The penetration of UV-B radiation is limited to the epidermis of window glass. Still, UV-A radiation can penetrate ordinary and colored glass and the epidermis [115]. When administered in low concentrations, UV-B radiation has been found to induce DNA damage, lymphocyte apoptosis, and the production of pro-inflammatory cytokines, adhesion molecules, and nitric oxide synthase in keratinocytes [116-118]. This might potentially have systemic ramifications. The transformation of trans- to cis-urocanic acid produced by UV-B radiation has been observed to inhibit cell-mediated immunity. Furthermore, UV-B radiation reduces Langerhans cells' ability to stimulate CD4+ Th1 cells. It activates CD4+CD45RA+ suppressor-inducer T-cells, thereby altering the immunological landscape in favor of B-cell activation [117,119]. UV-A radiation has the potential to induce systemic flares by directly infiltrating the subcutaneous vasculature. Conversely, it has been suggested that UV-A1 light with a longer wavelength range of 340-400 nm may possess protective properties against flare-ups in SLE [120]. Instances of organ involvement, including lupus nephritis, have been found in individuals diagnosed with SLE due to exposure to sunlight [121]. Interestingly, there are anecdotal reports of NPSLE after sunlight exposure in individuals with NPSLE [122].

Pharmaceutical compounds linked to drug-induced lupus erythematosus (DILE) have diverse chemical compositions, including aromatic amines, hydrazine, and sulfhydryl groups. This suggests that DILE does not possess a singular unifying chemical configuration [123]. Pharmacological agents implicated in DILE can be classified into four distinct categories, which include drugs that are definitive, likely, potentially, and recently documented to induce DILE [124]. The medications most frequently associated with DILE include hydralazine, procainamide, isoniazid, and TNF-α inhibitors [125]. Hydrochlorothiazide, calcium channel blockers, and angiotensin-converting enzyme inhibitors are among the drugs most prone to inducing subacute cutaneous lupus erythematosus (SCLE) [126]. Other medications like proton-pump inhibitors (PPIs), terbinafine, immunomodulators (leflunomide), TNF-κ inhibitors, and chemotherapeutic agents have the potential to elicit SCLE. Grönhagen et al. found higher odds ratios (OR) for terbinafine (OR 52.9), TNF-κ inhibitors (OR 8.0), antiepileptics (OR 3.4), and PPIs (OR 2.9) and SCLE [127-132]. Fluorouracil substances or their contemporary counterparts, such as capecitabine, have traditionally been identified as the primary triggers of chronic cutaneous DILE [133].

Spectrum of neurological manifestations in SLE

The ACR developed criteria for NPSLE, including 19 manifestations. These are classified into 12 central and seven PNS manifestations [15]. NPSLE may develop as a presenting symptom or throughout the disease course [15]. Neuropsychiatric syndromes occur in 56.3% of SLE patients [14]. Furthermore, their prevalence varies according to the age at which the disease begins. Pamuk et al. showed that seizures and psychosis were more common in early-onset SLE patients, while peripheral neuropathy was more prevalent in the late-onset SLE group [134].

Psychiatric Disorders (Anxiety, Depression, and Psychosis)

Mood disorders are prevalent in around 20% of patients with SLE [14]. The severity of depression and anxiety is associated with SLE activity [135]. Moreover, depression can affect drug adherence [136], leading to frequent SLE flares and a worse prognosis [137]. Psychosis is considered rare, occurring in 2.5% of patients with SLE [138]. Psychosis is a disorder in the perception of reality; patients with psychosis may experience hallucinations or delusions [15]. In around 5% of patients with SLE, psychosis may be caused by steroid administration [139].

In the realm of control and treatment of anxiety, depression, and psychosis, psychotherapy, occupational therapy, and psychoeducation are all highly effective methods. Pharmacotherapy and psychotherapy are currently the first-line treatments for depression in the United States. However, studies have shown that all modes of exercise (strength, aerobic, flexibility, and mind-body) are non-inferior to these treatments for mild to moderate depression [140]. Occupational therapists focus on adapting to the client’s physical and social environment. Modifications to the physical environment, such as ramps, stair lifts, and wheelchair-accessible showers, help promote confidence and self-reliance [141]. Psychoeducation involves providing information to family members to help them understand aspects of living with an illness so they can assist the patient and help reduce anxiety, especially in adolescents [142]. SLE-related psychosis can be managed with steroids and immunosuppressive drugs, which result in full remission for about 66.7% of patients [138]. Meanwhile, rituximab has shown significant improvement in refractory cases [143].

Cognitive Dysfunction

The ACR defines cognitive dysfunction as impairment in cognitive domains, including attention, memory, reasoning, and language [15]. Cognitive dysfunction is present in 19.7% of SLE patients [14]. However, it is often poorly recognized by physicians [144]. Cognitive dysfunction is not correlated with the severity of the disease but is associated with organ damage. Moreover, anti-dsDNA antibodies are negatively related to cognitive impairment [144]. The ACR has recommended a one-hour battery test for diagnosing cognitive impairment [15]. Psychosocial education has been shown to improve memory and functioning [145]. However, memantine did not significantly improve cognitive impairment in NPSLE [146].

Seizures

Seizures affect approximately 9.9% of SLE patients. The generalized tonic-clonic seizure is the most common seizure type in SLE patients [147]. Females and patients who develop SLE at a young age are at a higher risk of developing seizures related to SLE [147]. There is no association between autoantibodies, including lupus anticoagulant and anticardiolipin, and the risk of developing seizures [147]. The outcome of seizures related to SLE is generally favorable. Most seizures resolve without the need for long-term medications or significantly affecting the quality of life [147].

Stroke and TIAs

Cerebrovascular diseases are prevalent among 8% of SLE patients [14]. The typical risk factors for cerebrovascular diseases include diabetes, hypertension, and dyslipidemia [148]. Other risk factors include cutaneous vasculitis, anticardiolipin antibodies, and lupus anticoagulant antibodies [149]. Moreover, the APS is an important risk factor, leading to a hypercoagulable state [150]. This is done by inhibiting protein C, reducing prostacyclin, activating platelets, and activating endothelial cells [151]. TIAs are reversible neurological events. However, they are considered important stroke predictors in SLE patients [148].

Peripheral Neuropathy

Polyneuropathy occurs in 3% of SLE patients, while mononeuropathy occurs in 1.5% [14]. Jasmin et al. reported that sensory neuropathy was the most prevalent type among SLE patients [151]. Mahran et al. showed that neuromuscular ultrasound could be used in addition to nerve conduction studies in patients with SLE and suspected neuropathy, as it provided additional information on the pathophysiology of nerve involvement [152]. GBS is a rare presenting symptom in around 1.3% of SLE patients [153]. However, its incidence has been increasing. Twenty-seven cases of GBS associated with SLE have been reported in the literature [154]. These patients, aged 20-60, were 77% female. They were managed with various immunosuppressive drugs, including steroids and cyclophosphamide, with 63% achieving complete recovery [154]. However, it is essential to differentiate between GBS as a symptom of SLE and pure GBS co-occurring with SLE, as cyclophosphamide is ineffective in patients with pure GBS [155].

Demyelinating Syndromes

DSs are rare, occurring in around 0.3% of SLE patients [14]. The manifestations of DS include vision loss, diplopia, lower limb weakness, cranial nerve palsy, nystagmus, cognitive impairment, and an acute confusional state [156]. Differentiating SLE from multiple sclerosis (MS) can be challenging in clinical practice. However, certain manifestations can help guide the diagnosis of SLE. These include the presence of a rash, arthralgia, myalgia, and renal involvement [157]. Furthermore, the involvement of the PNS may suggest SLE, as MS only affects the CNS [158].

The pathology of MS differs from that of SLE. Plaques and focal areas of demyelination, inflammatory changes, and axonal degeneration characterize MS. In contrast, the pathology of SLE-related DS is associated with cerebrovascular disease, resulting in multiple areas of infarction, atherosclerosis, and angiopathy [158]. However, Nikolopoulos et al. reported that patients with SLE-related demyelinating diseases (SLE-DS) and those with SLE fulfilling the criteria for MS had similar neurological findings on MRI. Additionally, they had similar rates of optic nerve involvement. Patients with SLE-DS, however, had low odds of achieving an elevated IgG index. None of the SLE-DS patients had positive type II oligoclonal bands [159].

Diagnostic challenges in neurological SLE

Neurological and psychiatric symptoms are collectively known as NPSLE. The reported prevalence of NPSLE ranges from 14% to 95% and is more common in children than adults [11,12]. Diagnosing neurological SLE is challenging due to the complexity and variability of its manifestations. There are no specific criteria to diagnose NPSLE; based on the diagnosis of exclusion, less than 40% of neuropsychiatric symptoms will be attributed to SLE-induced nervous system damage. Other causes (e.g., drug-induced, primary disorders) better explain these symptoms in the remaining cases. Therefore, the physician must exclude other causes and assess neurological and psychiatric symptoms. Further evaluation includes assessing general SLE activity, cardiovascular risk factors, atherosclerotic disease, and thrombotic events [14,82].

Neurological assessment should focus on headaches, signs of seizures, and motor and sensory deficits, while psychiatric evaluation should assess behavior, cognition, perception, thinking, mood, and affect. Diagnosing patients with neurological manifestations in SLE should involve all the investigations typically conducted for non-SLE patients presenting with similar symptoms and signs [82]. For example, a patient with stroke symptoms should undergo a screening echocardiogram, vessel imaging of the carotid and vertebral arteries, and testing for SLE-specific causes such as aPL antibodies [160]. Another example is when a patient presents with a psychiatric disorder, such as confusion. The physician should identify the cause, whether it's metabolic abnormalities, infection, or psychoactive drug use.

Several studies have investigated a correlation between disease activity and neuropsychiatric events in SLE patients. Some studies have demonstrated a connection between heightened overall SLE disease activity and neuropsychiatric manifestations attributed to the disease. This correlation is stronger for diffuse rather than focal events [23]. Neuroimaging and laboratory tests are integral to diagnosing neurological SLE, particularly distinguishing its manifestations from other neurological conditions. The table below provides examples of investigations used for diagnosis (Table 2).

**Table 2:** Diagnostic tests for neurological SLE SLE: systemic lupus erythematosus, CNS: central nervous system, MRI: magnetic resonance imaging, CT: computed tomography, PET: positron emission tomography, CSF: cerebrospinal fluid, WM: white matter, SPECT: single-photon emission computed tomography, apL: antiphospholipid, NMO: neuromyelitis optica, NPSLE: neuropsychiatric systemic lupus erythematosus, MS: multiple sclerosis

References	Test	Utility in neurological SLE
Kozora et al. (1998) [161], Nomura et al. (1999) [162], Magro-Checa et al. (2016) [19]	MRI	The imaging technique of choice is MRI, especiallyT2-weighted images. The most frequent pathological pattern is small punctate hyperintense T2-weighted focal lesions in subcortical and periventricular WM, usually in the frontal-parietal regions. Unfortunately, these MRI lesions are also present in many patients without neuropsychiatric manifestations. MRI can exclude MS, malignancy, infarction, and subarachnoid hemorrhage.
Magro-Checa et al. (2016) [19]	CT	Used to exclude other causes of neurological symptoms such as bleeding, tumors, and hemorrhage.
Sibbitt et al. (1999) [163], Waterloo et al. (2001) [164]	PET	SPECT imaging has detected widespread and localized deficits in patients with SLE, which may be either persistent or reversible. SPECT imaging can be abnormal in up to half of patients with SLE with no clinical manifestations of neuropsychiatric disease. Thus, SPECT findings are not unique to SLE.
Zirkzee et al. (2014) [165], Birnbaum et al. (2007) [166], Birnbaum et al. (2009) [167]	Autoantibody profiling	Several circulating autoantibodies like apL antibodies and β2-glycoprotein antibodies correlate with disease activities, specifically focal events such as cerebrovascular disease and seizures. Antiribosomal P antibodies were found to be specifically related to lupus psychosis. Aquaporin 4 autoantibodies can help diagnose a patient with myelopathy, optic neuritis, and NMO.
Kozora et al. (1998) [161], Nomura et al. (1999) [162]	CSF analysis	Helps to exclude CNS infection in patients with fever or other signs and symptoms suggestive of infection; mild CSF abnormalities are common (40–50%) but are not specific to the NPSLE. It can exclude infection, malignancy, myasthenia gravis, and oligoclonal bands.
González-Duarte et al. (2008) [168], Appenzeller et al. (2004) [169]	CSF analysis	Helps to exclude CNS infection in patients with fever or other signs and symptoms suggestive of infection; mild CSF abnormalities are common (40–50%) but are not specific to the NPSLE. It can exclude infection, malignancy, myasthenia gravis, and oligoclonal bands.
Hanly et al. (2014) [170], Zirkzee et al. (2012) [171]	Neuropsychological testing	It’s carried out when suspicion of impaired cognitive abilities is present. Patients with suspected impaired cognitive ability should be referred for full neuropsychological assessment.

Management and treatment strategies for neurological SLE

The primary goal in managing neurological SLE is to control SLE disease activity, minimize neurological symptoms, and improve overall function and quality of life. Due to the diverse clinical presentations and difficulties in diagnosis, managing patients with SLE who experience neuropsychiatric symptoms is most effective when approached by a multidisciplinary team coordinated by a rheumatologist. Identifying and treating non-SLE-related factors in all cases is essential, even when SLE is the primary contributor. For instance, infections or metabolic issues must be managed in patients presenting with acute neuropsychiatric symptoms, while those with vascular-related neuropsychiatric events should be evaluated for cardiovascular risk factors. Specific therapies for primary SLE manifestations are chosen based on the predominant immunopathogenic mechanism in NPSLE, whether the injury is inflammatory-mediated or vascular-mediated.

When the etiology is thought to be inflammatory/neurotoxic (especially in cases of aseptic meningitis, optic neuritis, transverse myelitis, peripheral neuropathy, refractory seizures, psychosis, and acute confusional state) and in the presence of lupus activity, management includes glucocorticoids alone or in combination with other immunosuppressants such as azathioprine or cyclophosphamide [172]. In cases of severe symptoms refractory to standard therapy, plasma exchange [173], intravenous immunoglobulin [174], and rituximab (an anti-CD20 monoclonal antibody) have been used [175]. A randomized controlled trial by Barile-Fabris in 32 patients with acute, severe NPSLE reported a significantly better response to therapy with intermittent intravenous cyclophosphamide than with intravenous methylprednisolone (95% vs. 54%, p<0.03) [176]. In patients with APS, anticoagulation therapy is more effective than antiplatelet therapy for secondary prevention of arterial events such as stroke and TIA [177]. For patients with recurrent thrombosis while on warfarin, adjuvant therapies are added to their treatment plans, such as antiplatelet agents, antimalarial agents, and statins [178].

Antidepressants, anxiolytics, and antipsychotic agents are prescribed according to their standard indications in psychiatric disorders. At the same time, antiseizure therapy is initiated when high-risk features are present, such as serious brain injury, brain structural abnormalities (MRI), focal neurological signs, and epileptiform discharges [179].

Antimalarial drugs (hydroxychloroquine and chloroquine) are used in the primary prevention of SLE symptoms, especially for cutaneous and musculoskeletal involvement [82], and to reduce mortality [180]. Although no studies specifically address their effect on NPSLE symptoms, a preventive role of these drugs in CNS lupus has been suggested, particularly in cerebrovascular disease [181]. As shown in Table 3, immunosuppressants and biological therapy are the main lines of treatment for NPSLE.

**Table 3:** Management of neuropsychiatric manifestations in SLE SLE: systemic lupus erythematosus, DNA: deoxyribonucleic acid, IV: intravenous, CNS: central nervous system, NPSLE: neuropsychiatric systemic lupus erythematosus, IL-2: interleukin-2, GI: gastrointestinal, TPE: therapeutic plasma exchange, IVIG: intravenous immunoglobulin

References	Drug name	Mechanism of action	Indication	Administration	Side effects
Schäcke et al. (2002) [182], Bertsias et al. (2010) [82], Buttgereit et al. (2002) [183]	Corticosteroids	Modulate immune response and inflammation via glucocorticoid receptors	First-line drug used to control SLE flares (mild-severe)	IV methylprednisolone (1 g/day x 3 days), oral prednisolone (1 mg/kg/day) tapering over 3-12 months	Hypertension, dyslipidemia, osteoporosis, diabetes, cataracts, glaucoma psychiatric issues (10% risk), infections, and peptic ulcer disease
Hejaili et al. (2003) [184], Houssiau et al. (2010) [185], Ognenovski et al. (2004) [186]	Cyclophosphamide	Impairs DNA replication and immune cell proliferation. It is a prodrug that is converted by liver cytochrome 450 enzymes to its metabolite 4-hydroxy cyclophosphamide	Severe NPSLE manifestations, mainly CNS involvement	Monthly IV regimen (0.75–1.5 g/m²), 500 mg fixed dose in less severe cases	Alopecia, nausea, leukopenia, hemorrhagic cystitis, cardiotoxicity, gonadal failure
DiPiero et al. (2015) [187], Oelzner et al. (1996) [188]	Azathioprine	Inhibits purine synthesis, affects cellular and humoral immune functions	Used as maintenance therapy. In prevention of flares. It is the first option in mild NPSLE symptoms as a glucocorticoid-sparing agent	Oral (2–3 mg/kg/day)	Bone marrow suppression, hepatotoxicity, increased risk of infection
Allison et al. (2005) [189], Pisoni et al. (2005) [190]	Mycophenolate mofetil	Inhibits lymphocyte proliferation via inosine-5'-monophosphate dehydrogenase	Maintenance after induction in renal SLE the efficacy of this drug in NPSLE patients is very modest and lacks strong evidence for its efficacy in neuropsychiatric symptoms	Oral (1000–3000 mg/day)	GI intolerance, bone marrow suppression, infections
Zhou et al. (2008) [191], Wang et al. (2014) [192]	Methotrexate	Methotrexate is a folic acid antagonist. It inhibits IL-2 transcription, suppresses T-cell activity	Limited evidence for NPSLE; some reports suggest positive effects with intrathecal administration	Oral, subcutaneous, or intrathecal in CNS involvement	GI symptoms, stomatitis, increased liver enzyme levels, and mild cytopenia. In severe cases, it can cause liver fibrosis, interstitial pneumonitis, and severe pancytopenia
Bambauer et al. (2000) [193], Yang et al. (2014) [194]	Cyclosporin A	Inhibits IL-2 transcription, suppresses T-cell activity	Rarely used in NPSLE with Limited evidence, but may aid in organic brain syndrome and psychosis when combined with TPE	Oral (2.5–3 mg/kg/day), sometimes with plasma exchange	Hypertension, renal dysfunction, hypertrichosis
Glennie et al. (2007) [195], Tokunaga et al. (2007) [143]	Rituximab	Rituximab is a chimeric monoclonal antibody directed against the B-cell-specific antigen CD20	It is used in severe refractory NPSLE, but it lacks long-term data	IV (1,000 mg doses separated by 15 days)	Infusion reactions, infections
Camara et al. (2014) [196], Milstone et al. (2005) [174]	IVIG	Mixture of natural IgG antibodies derived from the blood of healthy donors. It inhibits the activity of autoreactive B lymphocytes, suppresses type I interferon-driven differentiation of dendritic cells, and reduces nucleosome endocytosis	Positive effects in small case reports with limited evidence. But, it is used as an adjunctive therapy in refractory cases	IV administration 2g/kg, with divided doses over 2–5 days	Mild and self-limited symptoms include headaches, fever, flushing, chills, arthralgia, and myalgia. Infusion reactions, risk of thrombosis

As shown in the previous table, corticosteroids are the first-line treatment for acute symptoms, especially during flares, but they are associated with significant long-term risks. Cyclophosphamide is preferred in severe cases due to its efficacy despite its side effects. Azathioprine and mycophenolate mofetil are used for maintenance therapy, although their role in managing neuropsychiatric symptoms is not well-studied. Rituximab offers the potential for refractory cases.

In the case of ischemic NPSLE, the primary treatments are anticoagulants and antiplatelets. Platelet activation is increased in SLE patients, and the presence of aPL antibodies adds additional risk for thrombosis [197]. It is recommended to use low-dose acetylsalicylic acid as primary thromboprophylaxis for SLE in patients who have positive lupus anticoagulant or persistently elevated anticardiolipin antibodies at medium to high levels [198].

For secondary prevention in patients with aPL antibodies, acetylsalicylic acid monotherapy, clopidogrel 75 mg monotherapy, and the combination of acetylsalicylic acid and extended-release dipyridamole are acceptable options for initial treatment [199,200]. On the other hand, anticoagulation therapy is preferred for secondary prevention in high-risk patients, as it is more effective at preventing stroke recurrence. However, the bleeding risk is higher, necessitating close monitoring with the international normalized ratio to ensure that the benefits of preventing thrombosis outweigh the risk of hemorrhagic complications. Sometimes, a combination of antiplatelet and anticoagulation therapies is used [201].

Prognosis and long-term outcomes in NPSLE

NPSLE is associated with a negative impact on quality of life and severe fatigue, regardless of the level of disease activity [202]. Moreover, patients with SLE are at increased risk of work incapacity compared to normal individuals [203]. Up to 34% of patients with SLE are unable to work. This is attributed to many factors, including socioeconomic status, disease activity, pain, fatigue, anxiety, and neurocognitive disorders [204]. Furthermore, cognitive impairment negatively impacts quality of life and participation in social activities [205].

Patients with NPSLE have an increased risk of mortality and morbidity compared to the general population. Li et al. reported an 11-fold increase in the risk of death [68]. The leading causes of death were related to NPSLE and involved increased intracranial pressure, cerebrovascular disease, and motor neuron disorders [68]. On the other hand, a focal CNS lesion is associated with a sevenfold increase in mortality [206]. Furthermore, the state of acute confusion, the level of C-reactive protein, and increased intracranial pressure are associated with mortality [68]. Also, receiving the appropriate treatment could affect the prognosis. Patients with neuropsychiatric events attributed to SLE who received steroids and immunosuppressive drugs had improved outcomes [207].

Many biomarkers have the potential to predict the prognosis of patients with SLE. Kostopoulou et al. showed that low levels of complement C3 and high levels of anti-dsDNA were associated with SLE flares [208]. Moreover, the presence of certain biomarkers could predict specific neuropsychiatric manifestations. Jiang et al. reported an association between psychosis and anti-β2-GPI antibodies, polyneuropathy and anti-Scl70 antibodies, and DS and anti-cardiolipin antibodies. Meanwhile, anti-Sjogren syndrome antigen B antibodies and high complement C3 were associated with a low risk of NPSLE [209]. Additionally, the prognosis of NPSLE could be predicted. Low C4 levels, lupus anticoagulant, and anti-cardiolipin antibodies were associated with severe neuropsychiatric manifestations [210,211]. Moreover, the treatment response could also be predicted. Zheng et al. showed that patients with low C3 or C4 levels or positive anti-dsDNA had better outcomes following belimumab administration [212].

For patients with SLE, there is a need for regular follow-up to monitor disease activity and treatment effects. Hsu et al. reported a case of GBS that was regularly followed up for a year after complete recovery [213]. This is critical because patients may not respond to treatment or experience a relapse. For example, in the case of demyelinating diseases, approximately 42% of patients with SLE and DS experienced a relapse [159]. In GBS associated with SLE, only 63% of patients experienced complete recovery [154]. Meanwhile, refractory symptoms could be managed with other approaches. Tokunaga et al. reported that patients with refractory NPSLE significantly improved following cyclophosphamide administration [143]. Moreover, SLE activity should be monitored as it could be associated with the severity of NPSLE [135].

Future directions of neurological SLE research

Numerous multinational initiatives have recorded advancements in SLE clinical management in recent years. The 2019 classification by the European League Against Rheumatism/American College of Rheumatology (EULAR/ACR) established positive antinuclear antibodies as a mandatory entry criterion, organized items into weighted organ domains, and substituted individual exclusion criteria with a singular attribution rule, stipulating that items should only be considered if no more plausible explanation than SLE exists. The revised EULAR recommendations explicitly outline significant advancements in SLE therapy, emphasizing the administration of hydroxychloroquine to all SLE patients without contraindications, the necessity of risk factor modification, treatment aimed at specific targets, and the reduction of glucocorticoid exposure. On the other hand, EULAR indicates that neuropsychiatric manifestations in SLE patients should be initially evaluated and treated as in patients without SLE and then ascribed to SLE and treated correspondingly. According to the type of manifestation, the diagnosis may involve lumbar puncture and CSF analysis, electroencephalogram, neuropsychological assessment of cognitive function, nerve conduction study, and MRI (including conventional MRI sequences, diffusion-weighted imaging, and gadolinium-enhanced T1 sequences) to evaluate brain structure and function [214]. The Latin American Grupo Latino Americano de Estudio del Lupus recommendations conveyed the same perspectives concerning antimalarials for all SLE patients and advocated for maintaining modest glucocorticoid dosages.

**Figure 1:**
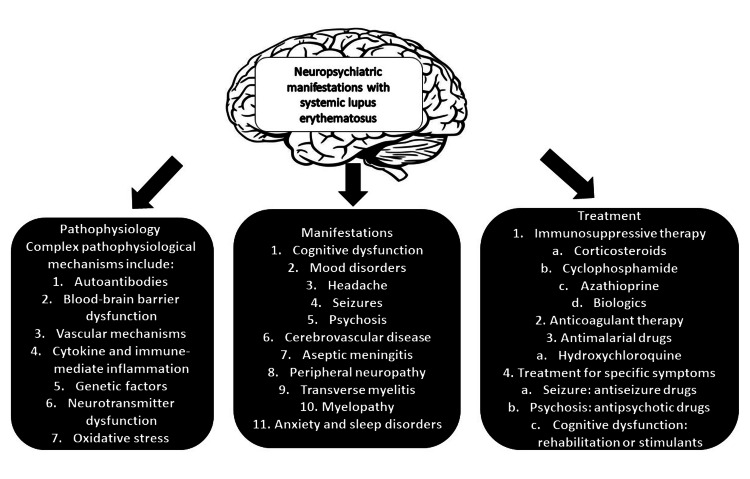
Pathophysiology, clinical manifestations, and treatment of NPSLE NPSLE: neuropsychiatric systemic lupus erythematosus Image Credit: Authors

SLE is a frequently observed disease in clinical practice, but the neurological symptoms related to this rheumatologic disorder are still poorly understood (Figure 1). There is an urgent need for studies involving this specific group of patients because they have a significantly worsened quality of life when compared to individuals without neuropsychiatric manifestations. Therefore, the development of biomarkers specific to NPSLE is mandatory. Also, the current biomarkers should be evaluated with meta-analysis regarding their benefit in diagnosing and assessing disease activity. As a general statement, a prompt diagnosis and treatment of NPSLE will likely result in a better prognosis.

Through further study of the procedure of immune abnormalities corresponding to active SLE, new therapies are being expanded in addition to identifying new targets for treating active disease. On the contrary, no new potential agents are available for NPSLE. To assess and design an effective intervention for NPSLE, understanding the pathophysiologic mechanisms causing it is necessary [215]. Clarification of some difficult clinical situations is also required. For example, the mechanisms involved in NPSLE are not well understood yet. Also, we face limitations in diagnostic tools because no in vivo imaging biomarkers present direct evidence for BBB dysfunction [216]. Moreover, randomized controlled trials (RCTs) to assess definitive treatment for NPSLE are limited, which leads to treatment methods being based on small controlled trials and expert recommendations. Lastly, in clinical settings, the cooperation of psychologists and neurologists is needed for diagnosing and treating NPSLE [217].

In the next 10 years, combining RCTs, NPSLE pathophysiological processes, and new strategies and methods may be beneficial for treating NPSLE patients. The clinical importance of symptom combination models needs to be specifically studied [217].

## Conclusions

NPSLE encompasses a diverse range of neurological manifestations that can significantly affect the quality of life of patients and, in many cases, result in long-term or even permanent disability. The spectrum of NPSLE includes both CNS and PNS involvement, with symptoms varying from cognitive dysfunction and mood disturbances to seizures and strokes. The pathophysiology of NPSLE is intricate and multifactorial, involving autoimmune mechanisms where autoantibodies target neural tissues, leading to neuroinflammation. Dysregulation of the complement system and genetic factors also play a pivotal role in developing these neurological complications. Despite the complexity of NPSLE, early detection and diagnosis remain critical. Prompt recognition of symptoms and appropriate diagnostic testing can help identify NPSLE before irreversible neurological damage occurs. Timely treatment interventions can significantly improve patient outcomes, reduce the severity of neurological impairments, and prevent permanent disability. Given the challenges associated with diagnosing NPSLE due to the overlap of symptoms with other conditions and the wide range of neurological manifestations, raising awareness among clinicians and fostering multidisciplinary collaboration are key to optimizing patient care.
